# Impact of Physical Therapy on Pain and Function in a Patient With Scoliosis

**DOI:** 10.7759/cureus.15261

**Published:** 2021-05-26

**Authors:** Vrushali Athawale, Pratik Phansopkar, Palak Darda, Neha Chitale, Ashvini Chinewar

**Affiliations:** 1 Musculoskeletal Physiotherapy, Ravi Nair Physiotherapy College, Datta Meghe Institute of Medical Sciences, Wardha, IND

**Keywords:** scoliosis, physical therapy, rehabilitation, spine deformity, cobb’s angle

## Abstract

Human spine is a complex and robust structure. Almost all spine deformities contribute to limitations in activities of daily living. Scoliosis is the most common deformity accompanied by rotation and progresses during the growth of an individual. It is classified into three categories: congenital, idiopathic, and neuromuscular. The common secondary causes of scoliosis include cerebral palsy, poliomyelitis, and other neuromuscular conditions. A case of a 23-year-old female with right shoulder pain with a history of adolescent idiopathic scoliosis, which leads to a decrease in self-image and disturbance in activities of daily living, is presented in this report. The assessment, medical history, and rehabilitation protocol are mentioned in this case report. Physical therapy to treat shoulder joints includes thermotherapy and manual therapy targeting pain over the joint and stiffness. The use of thermotherapy, bracing, and strengthening and stretching exercises to prevent further deformity and aggravation of the symptoms is described in this report. We report that there was a significant improvement in muscle strength, relief from pain, spinal mobility, postural control, and decreases in further complications.

## Introduction

The lateral curvature of the spine is scoliosis, which is commonly accompanied by rotation. The scoliosis curve progresses during the growth of the individual, resulting in functional deformity. The curvature gets manifested even before skeletal maturity, but early management of manifestation could lead to restricting the adverse effects before reaching skeletal maturity, whereas in untreated patients it can continue after skeletal maturity [1]. Scoliosis is classified as either congenital, idiopathic, or neuromuscular. Secondary causes of scoliosis are cerebral palsy, poliomyelitis, Werdnig Hoffman’s disease, developmental dysplasia of the hip, and syringomyelia [2]. The vertebral spine with scoliosis changes usually depicts an S line on X-ray and forms a complex three-dimensional deformity of the trunk and spine. The pathological symptoms are back pain, respiratory impairment in breathing, disability, poor perception of body image, and segmental instability [3]. On anteroposterior radiographs, the security of curvature is measured. Cobb’s angle determines the severity of lateral curvature and deformity [4]. Adolescent idiopathic scoliosis begins in adolescents in 89% of cases [5]. Surgical treatments for scoliosis include decompression, limited short fusion, and long fusion with correction of deformity [6]. Physical therapy plays a key role in preventing the scoliosis curve and deformity, and improving the outcomes and quality of life through manual therapy, bracing, core stabilization, and strengthening [7-9]. The case presented in this report is of adolescent idiopathic scoliosis with unilateral right side shoulder joint pain.

## Case presentation

A 23-year-old female with right-hand dominance was referred to the physiotherapy department. The patient stated that she had right shoulder pain radiating over the elbow joint, severe back pain while standing, and decreased self-image. Developmental milestone history was normal. She was able to sit at six months, crawl at seven months, stand at one year, and walk at one year three months. Her spine examination revealed scoliosis with the dropping of the left shoulder and a prominent inferior angle of the right scapula. The patient presented with dull aching pain at the anterior and posterior shoulder joint capsule, both insidious in onset, with dull aching aggravated by activities and relieved by rest with no radiation and aggravated at night. On the numerical pain rating scale (NPRS), she rated her pain as 6. She also complained of pain throughout her entire spine, which was aggravated by standing and walking. On the NPRS, she rated her pain as 7.

Clinical findings

**Figure 1:**
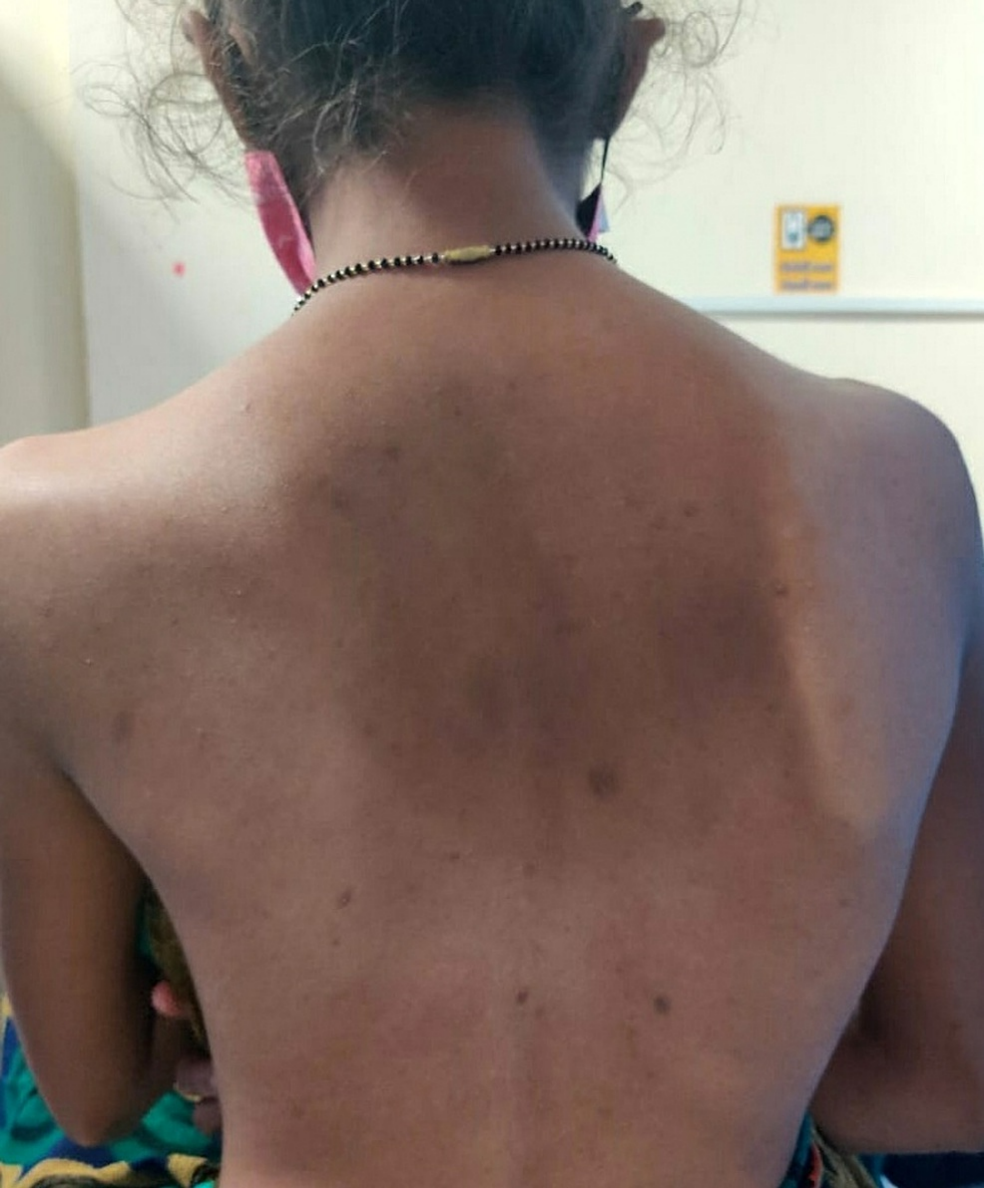
Posterior postural evaluation The scoliotic curve is noted, with drooping of the left shoulder and a prominent inferior angle of the scapula.

**Figure 2:**
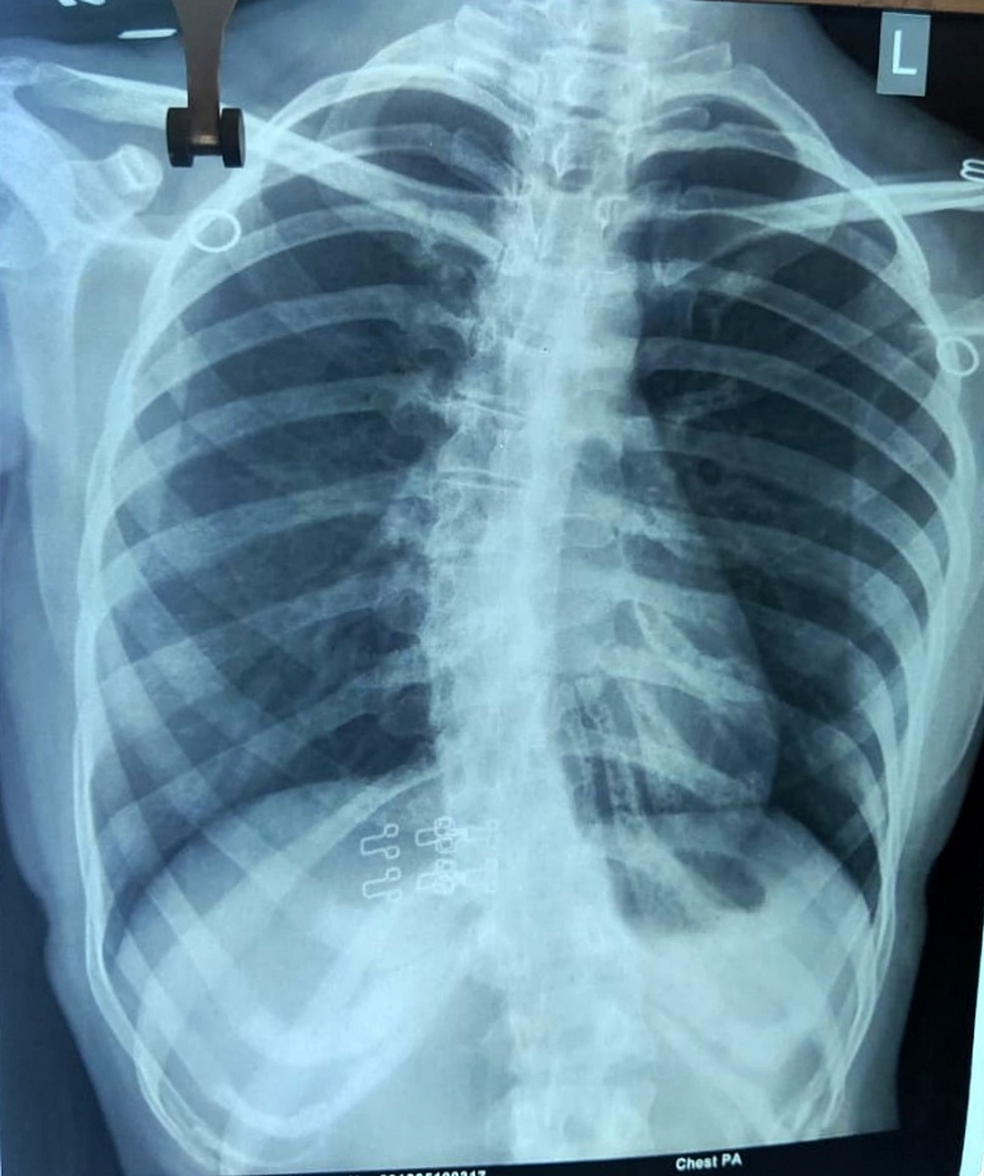
X-ray of the spine (posterior-anterior view) The scoliotic curve is noted with convex curvature on the left side and concave curvature on the right side.

**Figure 3:**
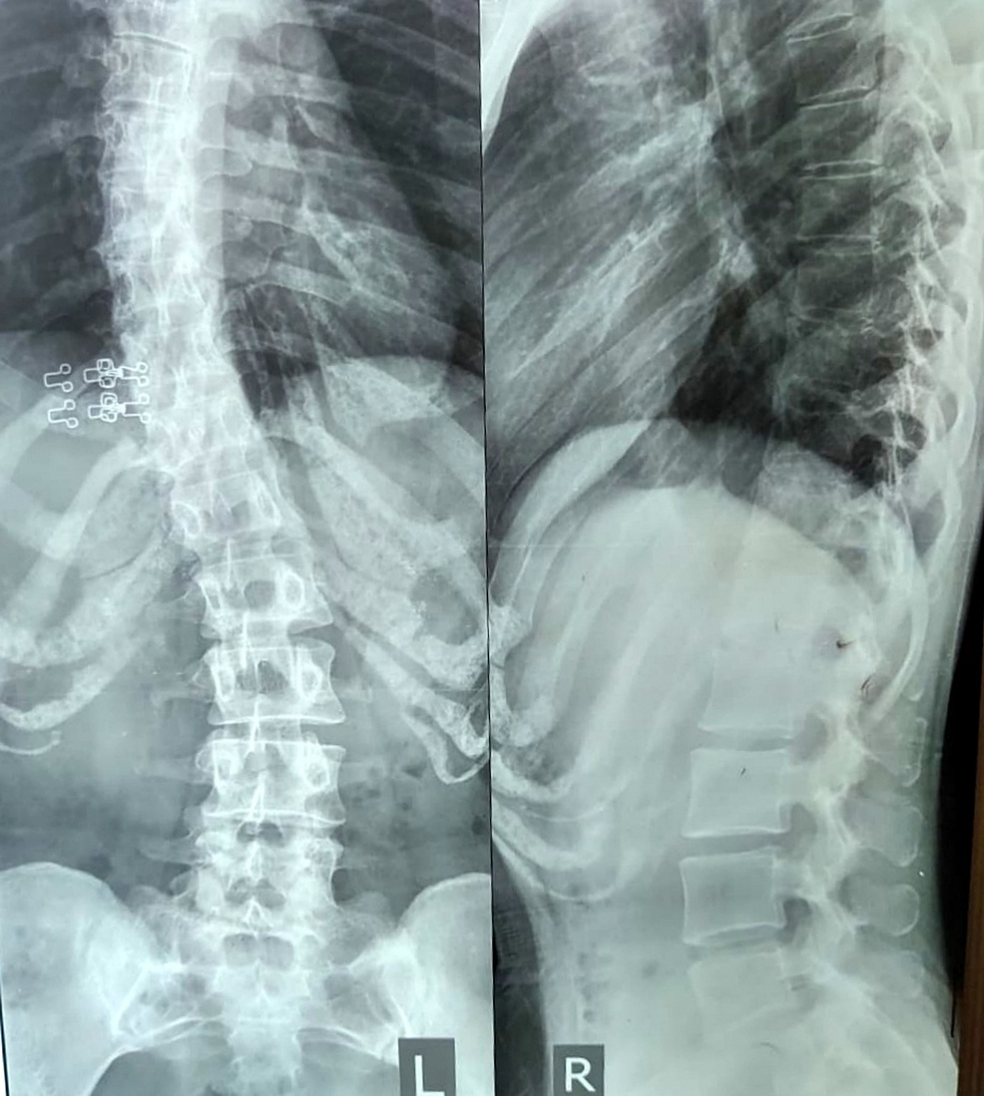
X-ray of the spine (anterior-posterior view and lateral view) The scoliotic curve is noted with tilting of the spinous process and reduced intervertebral space.

After obtaining consent from the patient, she was examined. Posture assessment was done initially in all planes. Drooping of the left shoulder was noticed (Figure 1) with the pelvis on the same side, and an S-shaped spine with the right-side prominent inferior angle of the scapula (Figures 2, 3). On palpation, there was no rise in local temperature. On the NPRS, she rated her right shoulder and low back pain as 8. On examination, chest expansion was mildly decreased posteriorly. Tenderness was present over the right posterior shoulder. Range-of-motion assessment was done by a goniometer (Tables 1, 2). Manual muscle tests were done with lumbar flexors-extensors, core abdominals, shoulder flexors, extensors grade 4+, abductors adductors grade 3+, internal-external rotators grade 4+, elbow flexors- extensors grade 4+, and wrist flexors-extensors grade 4+.

**Table 1:** ROM assessment of joint on the first day of rehabilitation ROM: range of motion

Joint	Active ROM (in degrees)	Passive ROM (in degrees)
Shoulder Flexion	0-160	0-170
Shoulder Extension	0-40	0-50
Shoulder Abduction	0-165	0-175
Elbow Flexion	0-140	0-140

**Table 2:** Range-of-motion assessment of spine on the first day of rehabilitation (by modified Schober method)

Movement	Measurement
Thoracolumbar Flexion	3 cm
Thoracolumbar Extension	1 cm
Thoracolumbar Lateral Flexion	3.5 cm

Therapeutic management

WEEK 1

Therapeutic interventions were started to reduce shoulder joint pain. Initially, a hydrocollator pack was applied to the shoulder joint for 10-15 minutes for relaxation and to reduce muscle spasm and pain. Interferential therapy was also started over the joint to reduce the pain for seven days, 10 minutes per session. Normal range-of-motion exercises of the upper extremity were actively taught to the patient.

WEEK 2

The patient was advised to use Milwaukee brace and instructed to wear it for 20 hours per day. The proper examination was held during brace treatment to consider any adverse effects (skin trouble, brace breakage, posture). The hydrocollator pack was applied to the back for 10-15 minutes. Myofascial release (MFR) techniques were applied directly to the skin without lotion or ointment. The pressure is applied over the restricted area for about 90-120 seconds to release the tissue, which helps to prevent back pain.

WEEK 3

The core stabilization exercises were started. The muscles involved are the diaphragm, multifidus, transverse abdominals, and the muscles around the pelvis. Pelvis tilt was started in a supine position with a 5-second hold for 10 repetitions, two times a day. Similarly, cat and camel, double-leg abdominal presses, and super woman were continued with the same protocol. Breathing exercises were also started, which included diaphragmatic breathing and thoracic expansion exercises for five repetitions twice a day. Proper bracing and MFR were also continued.

WEEK 4

The stretching exercise was started for the back and neck extensors, which included standing forward bending, modified slump, lateral bending in standing both sides, lateral flexion of the neck, and lateral rotations each carried out for 15 repetitions for three sets and progressed to 25 repetitions for three sets. Stretching core stabilization exercises and breathing exercises were also continued. Segmental breathing exercises were started.

WEEK 5

Scapular strengthening exercises were focused on the trapezius, serratus anterior, levator scapulae, and rhomboids. Exercises including shoulder shrugging, push-ups, diagonal proprioceptive neuromuscular facilitation, shoulder flexion-horizontal flexion-flexion-external rotation, and resisted neck isometric exercises with maximum resistance for 10-15 repetitions were continued once a day. Stretching exercises, core stabilization, and breathing exercises were continued with progression by increasing the repetitions, sets, and holds. Resistance was added with a segmental breathing pattern.

Follow-up and outcomes

After five weeks of physical therapy, there was an improvement in pain scores for the right shoulder joint and low back pain. Chest expansion was also improved. On NPRS, the right shoulder and low back pain were rated as 2. Clinically, the improvements noted were that the patient could stand for a longer duration than before with mild discomfort during the activities of daily living. The range of motion for the concerned upper limb and lumbar range of motion were also improved (Tables 3, 4). Manual muscle tests were done with lumbar flexors-extensors, core abdominals, shoulder flexors, extensors grade 5, abductors adductors grade 5, internal-external rotators grade 5, elbow flexors-extensors grade 5, and wrist flexors-extensors grade 5.

**Table 3:** ROM assessment of joint after five weeks of rehabilitation ROM: range of motion

Joint	Active ROM (in degrees)	Passive ROM (in degrees)
Shoulder Flexion	0-180	0-180
Shoulder Extension	0-50	0-50
Shoulder Abduction	0-180	0-180
Elbow Flexion	0-140	0-140

**Table 4:** Range-of-motion assessment of spine after five weeks of rehabilitation (by modified Schober method)

Movement	Measurement
Thoracolumbar Flexion	5.5 cm
Thoracolumbar Extension	2 cm
Thoracolumbar Lateral Flexion	6 cm

## Discussion

The patient complained of pain over the shoulder joint and back, a decrease in chest expansion, and lateral curvature of the spine. After clinical evaluation, a proper rehabilitation protocol was developed to decrease pain and prevent complications. Brace treatment plays a beneficial role in preventing further deformity. Milwaukee brace was used on the inside of the convex side in a bending position. Wearing a brace at night shows a more positive effect on the correction of the spine curve [10,11]. Thermotherapy was advised by applying hot packs, which increase temperature, blood flow, and cell metabolism and help in the healing process by removing cell debris, decreasing pain, and relaxing muscles [12]. The MFR technique has been indicated to decrease pain and to facilitate the restrictions of fascia. Gentle pressure was applied over the tightened structures. In our case report, this technique has been shown to provide a positive effect, releasing restricted structures of the back [13,14]. Core stabilization exercises are developed as exercise regimes for postural balance and they play a key role in trunk control in static and dynamic postures. In this case report, this approach has been shown to improve muscle imbalance and decrease pain and rotation deformity [15,16]. Pulmonary functions are affected in scoliosis and are painless and asymptomatic in the early stage, but can later cause complications. In our case, breathing exercises improved breathing control, increased inspiratory muscle strength and endurance, and reduced respiratory complications [17]. Stretching exercises are exercises where particular muscles, tendons, and muscle groups are stretched, which showed a positive effect in improving elasticity [18]. Scapular strengthening exercises specifically target the scapular muscles to improve strength. In our case report, all the factors essential for strengthening and maintaining posture in static and functional activities for a scoliotic patient are well presented, which can be beneficial for structuring a rehabilitation program for any patient with idiopathic scoliosis.

## Conclusions

Adolescent idiopathic scoliosis is the most common form of scoliosis and can be treated conservatively with proper rehabilitation protocol to prevent further deformity. Following five weeks of rehabilitation in this case, there was a significant improvement in posture, muscle strength, and pain, in turn preventing future complications. This case report establishes a properly structured and comprehensive rehabilitation protocol for dealing with scoliosis of the spine with unilateral shoulder joint pain.
